# Once-weekly Insulin Icodec Versus Once-daily Long-acting Insulin for Type II Diabetes: A Meta-analysis of Randomized Controlled Trials

**DOI:** 10.1210/jendso/bvad177

**Published:** 2023-12-28

**Authors:** Mohamed Abuelazm, Ahmed A Ibrahim, Yehya Khlidj, Amr Badr, Ahmed Mazen Amin, Mohamad A Elzeftawy, Ibrahim Gowaily, Ahmed Saad Elsaeidy, Basel Abdelazeem

**Affiliations:** Faculty of Medicine, Tanta University, Tanta 31111, Egypt; Faculty of Medicine, Menoufia University, Menoufia 32511, Egypt; Faculty of Medicine, Algiers University, Algiers 44002, Algeria; Department of Cardiology, Banha Teaching Hospital, Banha 13511, Egypt; Faculty of Medicine, Mansoura University, Mansoura 35516, Egypt; Faculty of Medicine, Tanta University, Tanta 31111, Egypt; Faculty of Medicine, Tanta University, Tanta 31111, Egypt; Faculty of Medicine, Banha University, Banha 13518, Egypt; Department of Cardiology, West Virginia University, Morgantown, WV 26505, USA; Department of Internal Medicine, Michigan State University, East Lansing, MI 48824, USA

**Keywords:** insulin, diabetes, glucose, review, analysis

## Abstract

**Background:**

Insulin icodec is a novel basal insulin analog with once-weekly subcutaneous administration. We aim to estimate the efficacy and safety of insulin icodec vs long-acting insulin (insulin glargine and degludec) in type II diabetic patients.

**Methods:**

We conducted a systematic review and meta-analysis synthesizing randomized controlled trials (RCTs), which were retrieved by systematically searching PubMed, Web of Science, SCOPUS, and Cochrane through May 29, 2023. We used RevMan V. 5.4 to pool dichotomous data using risk ratio (RR) and continuous data using mean difference (MD) with a 95% confidence interval (CI). Our primary outcome was glycated hemoglobin (HbA1C) change.

**Results:**

We included 7 RCTs with a total of 3183 patients. Insulin icodec was associated with significantly decreased HbA1C (MD: −0.15 with 95% CI [−0.24, −0.06], *P* = .002) and increased percentage of time with glucose in range (TIR) (MD: 4.06 with 95% CI [2.06, 6.06], *P* = .0001). However, insulin icodec was associated with increased body weight (MD: 0.57 with 95% CI [0.45, 0.70], *P* = .00001). Also, there was no difference regarding any serious adverse events (AEs) (RR: 0.96 with 95% CI [0.76, 1.20], *P* = .7) or AEs leading to withdrawal (RR: 1.54 with 95% CI [0.84, 2.82], *P* = .16). However, insulin icodec was associated with increased any AEs incidence (RR: 1.06 with 95% CI [1.01, 1.12], *P* = .02).

**Conclusion:**

Insulin icodec was associated with decreased HbA1C, increased TIR, with similar hypoglycemic and serious AEs. However, it was also associated with increased body weight and the incidence of any AEs.

Type II diabetes mellitus (T2D) is a chronic metabolic disorder characterized by hyperglycemia because of insulin resistance and insufficient compensatory insulin secretion [1]. Insulin was first developed as a therapeutic agent for diabetic patients in the 1920s. The initial formulas were injected several times a day because of their short half-lives. Since then, many attempts have been made to modify the feasibility of insulin medications by creating insulin preparations with longer half-lives to reduce daily dosages [2]. Insulin glargine and insulin degludec are long-acting basal insulins that are used to treat type I and II diabetes. Both drugs are injected once daily with long-lasting effect that helps to keep blood sugar levels stable throughout the day [3].

However, T2D patients on insulin therapy still face many challenges, such as treatment adherence and persistence [4]. Treatment nonadherence is due to multiple reasons, including prescribed injection phobia, complexity of dosing schedules, negative impact on daily life, concerns about long-term medication use, and insulin shots cost [5, 6]. Also, physicians may delay initiating insulin therapy because of the patient's fears of weight gain and hypoglycemic episodes associated with insulin therapy [7]. Moreover, severe hypoglycemia is associated with macro-vascular events and cardiovascular mortality among T2D patients on long-acting basal insulins [8].

Insulin icodec is a novel ultra-long basal insulin developed by Novo-Nordisk for once-weekly insulin administration in diabetic patients. It reaches the maximum plasma concentration 16 hours after administration in T2D patients. Also, insulin icodec is slowly and consistently released in circulation with a mean half-life of 196 hours because of its albumin-binding properties. Over the week, insulin icodec has a nearly uniformly distributed effect on reducing blood sugar levels, with a steady-state reached after 3 to 4 once-weekly dosing [9]. Also, T2D patients reported positive perspectives on once-weekly insulin therapy, which may improve their convenience, adherence, and quality of life [10]. Consequently, once-weekly insulin is expected to provide better glycemic control in T2D patients. In April 2023, Novo Nordisk submitted a biologics license application to the US Food and Drug Administration for once-weekly insulin icodec for T2D treatment based on the ONWARDS program, and the decision is expected in April of 2024 [11].

Multiple recent randomized controlled trials (RCTs) have investigated insulin icodec for T2D with some conflicting results [12-18]. When compared to once-daily insulin, insulin icodec has recently shown a similar absolute reduction in glycated hemoglobin (HbA1c) and hypoglycemic episodes. Also, more patients in the icodec group reached the HbA1c targets (7.0% and 6.5%), with patients in the insulin icodec group experiencing more time in the desirable glycemic range of 70 to 140 mg/dL than patients on long-acting insulin [13]. However, Mathieu et al reported more adverse events in the icodec group [15]. Therefore, this systematic review and meta-analysis aimed to synthesize the evidence from the recent RCTs, investigating the efficacy and safety of once-weekly insulin icodec vs once-daily long-acting insulin (insulin glargine and insulin degludec) in T2D patients.

## Methodology

### Protocol Registration

The protocol for this review has been registered and published in PROSPERO with the following ID: CRD42023434794. Our systematic review and meta-analysis were conducted guided by the PRISMA statement (Preferred Reporting Items for Systematic Reviews and Meta-analysis) [19] and the Cochrane Handbook for Systematic Reviews and Meta-Analyses [20].

### Data Sources & Search Strategy

B.A. and M.A. conducted a comprehensive search of the following electronic major databases: MEDLINE (PubMed), EMBASE, Scopus, Web of Science, and Cochrane Central Register of Controlled Trials (CENTRAL) up to June 2023. We applied no search filters or limits. The search strategy, including the results, is outlined in Supplementary Table S1 [21]. An updated manual PubMed search was conducted up to August 2023.

### Eligibility Criteria

We included RCTs that followed the following PICO criteria: population (P): patients with type II DM; intervention (I): insulin icodec; control (C): long-acting insulin (insulin glargine or insulin degludec); outcome (O): our primary outcome was the change in HbA1C, while secondary outcomes included time in range (TIR) blood glucose using continuous glucose monitoring, change in fasting plasma glucose (FPG), change in body weight, and safety outcomes (adverse and hypoglycemic events).

### Study Selection

Covidence online software was used to conduct the review process, which was performed independently by four reviewers (A.B., A.M.A., I.G., and M.A.E.), after removing duplicate records. The full texts of the potentially eligible records were screened against the previous eligibility criteria. Disagreements were resolved after involving B.A. in the discussion.

### Data Extraction

Four reviewers (A.B., A.M.A., I.G., and M.A.E.) independently extracted the following data using a pilot-tested extraction sheet: summary characteristics (study design, country, total participants, insulin icodec intervention details, control details, eligible HbA1C range, target fasting glucose, previous basal insulin therapy, main inclusion criteria, use of short-acting bolus insulin, insulin dose-adjustments, primary outcome, and follow-up duration); baseline characteristics (number of participants in each group, age, sex, body mass index, diabetes duration, HbA1C, fasting plasma glucose); and outcome data as previously described.

### Risk of Bias and Certainty of Evidence

Four reviewers (A.B., A.M.A., I.G., and M.A.E.) assessed the quality of the studies included in the research independently using the Cochrane ROB2 tool [22]. The domains that were evaluated included the risk of bias resulting from the randomization process, the risk of bias due to deviation from the intended intervention, the risk of bias due to missing outcome data, the risk of bias in measuring the outcome, and the risk of bias in selecting the reported results. In the event of any disagreements, the reviewers discussed and resolved them through consensus.

To appraise the quality of evidence, 2 reviewers (M.A. and B.A.) utilized the Grading of Recommendations Assessment, Development, and Evaluation (GRADE) guidelines [23, 24]. We considered inconsistency, imprecision, indirectness, publication bias, and risk of bias. The evaluation was carried out for each outcome, and the decisions made were justified and documented. Any discrepancies were settled through discussion.

### Statistical Analysis

The RevMan v5.3 program was used to carry out the statistical analysis [25]. To combine the results of dichotomous outcomes, we used the risk ratio; for continuous outcomes, we used the mean difference (MD), both with a 95% confidence interval (CI). To assess heterogeneity, we employed the Chi-square and I-square tests, where the Chi-square test assesses the presence of heterogeneity, and the I-square test assesses its degree. We interpreted the I-square test as follows: not significant for 0% to 40%, moderate heterogeneity for 30% to 60%, substantial heterogeneity for 50% to 90%, and considerable heterogeneity for 75% to 100%, following the Cochrane Handbook (chapter 9) [20]. We considered an alpha level below 0.1 for the Chi-square test to denote significant heterogeneity.

We used Trial Sequential Analysis (TSA) to assess the reliability and conclusiveness of the data from the pooled trials and to assess whether the sample size of the current meta-analysis was adequate to draw solid conclusions regarding the impact of the interventions. When the Z-line on the curve crosses both the conventional boundary and the trial sequential monitoring boundary, it is assumed that the intervention's confidence level is conclusive and sufficient, and no additional studies are required. On the other hand, if the Z-line does not cross any boundaries, the evidence needs to be more sufficient, and further studies are necessary [26, 27]. In this meta-analysis, we used an alpha error of .05, a beta error of 80% power, and an anticipated relative risk (RR) reduction of 20% in dichotomous outcomes.

## Results

### Search Results and Study Selection

**Figure 1.**
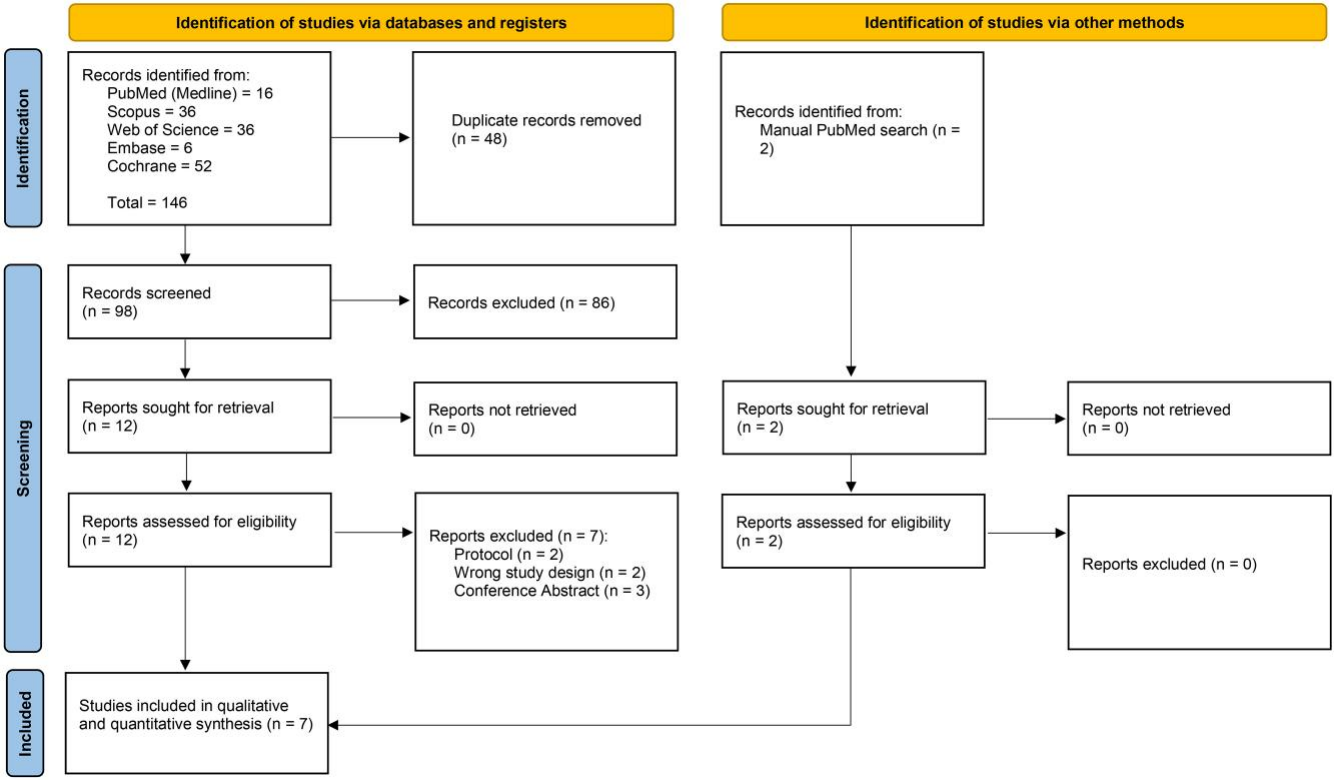
PRISMA flow chart of the screening process.

During the search process, a total of 146 studies were identified and evaluated for screening based on their titles and abstracts. After removing 48 duplicates and 86 studies that did not meet the inclusion criteria, 12 full-text articles were assessed. Out of these, 7 were found to be irrelevant and excluded, leaving a total of 5 RCTs. Moreover, we manually added 2 further RCTs through an updated PubMed search, leaving a total of 7 RCTs to be included in the qualitative and quantitative analysis (Fig. 1).

### Characteristics of Included Studies

A total of 7 RCTs [12-18] with 3183 participants were included in the final analysis. Three studies were phase II RCTs, and 4 studies were phase III RCTs. The mean age of the study population ranged from 58 to 62 years. Five studies had insulin glargine as the comparator group, while 2 studies had insulin degludec as the comparator group. Further summary details of the included RCTs and baseline data of the participants are outlined in Tables 1 and 2.

**Table 1.** Summary characteristics of the included RCTs

Study ID	Study design	No. of participants	Control	Eligible HbA1C range	Target fasting SMBG	Bolus dose of basal insulin	Previous basal insulin therapy	Use of short-acting bolus insulin	Insulin dose-adjustments	Primary outcome	Overall ROB
Bajaj et al, 2021	Multicenter, open-label phase II RCT	154	IGlar 100 U	7.0% to 10.0% (53.0-85.8 mmol/mol)	4.4 to 7.2 mmol/L (80-130 mg/dL)	Yes	Yes	Yes (Aspart only as a rescue medication during the trial	Icodec (±28 U/week); IGlar U100 (±4 U/day)	Time with glucose in range	Some concerns
Lingvay et al, 2021 (titration B group)	Multicenter, open-label phase II RCT	102	IGlar 100 U	7.0% to 10.0% (53.0-85.8 mmol/mol)	4.4 to 7.2 mmol/L (80-130 mg/dL)	No	No	No	Icodec (±28 U/week); IGlar U100 (±4 U/day)	Time with glucose in range	Some concerns
Lingvay et al, 2023 (ONWARDS 3)	Multicenter, open-label phase III RCT	588	Insulin degludec	7.0% to 11.0% (53-97 mmol/mol);	4.4 to 7.2 mmol/L (80-130 mg/dL)	No	No	No	Icodec (±20 U/week); Degludec (±3 U/day)	HbA1C change	Some concerns
Mathieu et al, 2023 (ONWARDS 4)	Multicenter, open-label phase III RCT	582	IGlar 100 U	7.0% to 10.0% (53-86 mmol/mol).	4.4 to 7.2 mmol/L (80-130 mg/dL)	No	Yes	Yes (Aspart 100 U/mL during the trial)	Icodec (±20 U/week); IGlar U100 (±3 U/day); Aspart (±1 U/day)	HbA1C change	Some concerns
Philis-Tsimikas et al, 2023 (ONWARDS 2)	Multicenter, open-label phase III RCT	526	Insulin degludec	7.0% to 10.0%	4.4 to 7.2 mmol/L (80-130 mg/dL)	Yes	Yes	No	Icodec (±20 U/week); Degludec (±3 U/day	HbA1C change	Some concerns
Rosenstock et al, 2020	Multicenter, double-blinded phase II RCT	247	IGlar 100 U	7.0% to 9.5%	3.9 to 6.0 mmol/L (70-108 mg/dL)	No	No	No	Icodec (±28 or ±14 U/week); IGlar U100 (±4 or ±2 U/day)	HbA1C change	Low
Rosenstock et al, 2023 (ONWARDS 1)	Multicenter, open-label phase III RCT	984	IGlar 100 U	7% to 11% (53.0 to 96.7 mmol/mol)	4.4 to 7.2 mmol/L (80-130 mg/dL)	No	No	No	Icodec (±20 U/week); IGlar U100 (±3 U/day)	HbA1C change	Some concerns

Abbreviations: HbA1c, glycated hemoglobin; IGlar, insulin glargine; RCT, randomized controlled trial; ROB, risk of bias; SMBG, self-measured blood glucose.

**Table 2.** Baseline characteristics of the participants

Study ID	Number of patients in each group		Age (Years), Mean (SD)		Sex (Male), n (%)		BMI, Mean (SD)		Diabetes duration (years), Mean (SD)		HbA1C (%), Mean (SD)		Fasting plasma glucose (mg/dL), Mean (SD)		Drop rates N. (%)		Race, N. (%)											
	Icodec	Control	Icodec	Control	Icodec	Control	Icodec	Control	Icodec	Control	Icodec	Control	Icodec	Control	Icodec	Control	Asian		Black or African-American		White		American Indian or Alaska Native		Native Hawaiian or Pacific Islander		other	
																	Icodec	Control	Icodec	Control	Icodec	Control	Icodec	Control	Icodec	Control	Icodec	Control
Bajaj et al, 2021 (LD group)	54	50	62.4 (7.2)	60.5 (7.9)	39 (72.2)	33 (66.0)	30.2 (4.3)	30.3 (5.0)	13.8 (7.7)	14.8 (8.1)	7.8 (0.7)	7.9 (0.7)	142 (34)	148 (36)			4 (7.4)	3 (6.0)	3 (5.6)	3 (6.0)	46 (85.2)	44 (88.0)	N/A	1 (1.9)	0	N/A	4 (7.4)	3 (6.0)
Bajaj et al, 2021 (NLD group)	50		62.1 (8.2)		39 (78.0)		29.0 (4.1)		16.8 (8.2)		7.9 (0.7)		144 (41)				9 (18.0)		2 (4.0)		39 (78.0)				9 (18.0)		2 (4.0)	
Lingvay et al, 2021 (titration B group)	51	51	61.2 (8.0)	60.2 (8.1)	54.9	52.9	31.4 (4.7)	30.6 (4.7)	9.6 (4.9)	11.8 (6.8)	8.1 (0.8)	8.2 (0.8)	180 (38)	168 (42)			80 (27.2)	85 (28.9)	9 (3.1)	6 (2.0)	179 (60.9)	175 (59.5)	0	1 (0.9)	N/A	11 (3.7)	11 (3.7)	80 (27.2)
Lingvay et al, 2023 (ONWARDS 3)	294	294	58 (10)	59 (10)	185 (62.9)	184 (62.6)	29.9 (5.2)	29.2 (5.1)	10.3(6.6)	11(7.3)	8.55 (1.11)	8.48 (1.01)	187 (54)	176 (46)			80 (27.2)	85 (28.9)	9 (3.1)	6 (2.0)	179 (60.9)	175 (59.5)	0	1 (0.9)	N/A	11 (3.7)	11 (3.7)	80 (27.2)
Mathieu et al, 2023 (ONWARDS 4)	291	291	59.7 (10.1)	59.9 (9.9)	154 (53)	150 (52)	30.5 (5.0)	30.0 (5.0)	18.0 (9.1)	16.3 (7.7)	8.29 (0.86)	8.31 (0.90)	167 (54)	173 (63)			N/A											
Philis-Tsimikas et al, 2023 (ONWARDS 2)	263	263	62.3 (9.8)	62.6 (8.4)	162 (62)	140 (53)	29.5 (5.2)	29.2 (4.9)	16.5 (8.4)	16.9 (7.9)	8.17 (0.77)	8.10 (0.77)	152.2 (47.5)	150.7 (40.9)			86 (33)	110 (42)	11 (4)	12 (5)	161 (61)	137 (52)	2 (1)	0	N/A	3 (1)	4 (2)	86 (33)
Rosenstock et al, 2020	125	122	59.7 (8.2)	59.4 (9.5)	70 (56.0)	69 (56.6)	31.1 (4.9)	31.4 (4.4)	10.5 (8.4)	8.8 (6.1)	8.09 (0.70)	7.96 (0.65)	180 (42)	180 (42)			N/A											
Rosenstock et al, 2023 (ONWARDS 1)	492	492	59.1 (10.1)	58.9 (9.9)	295 (60.0)	263 (53.5)	30.0 (4.8)	30.1 (5.1)	11.6 (6.7)	11.5 (6.8)	8.5 (1.0)	8.4 (1.0)	185.3 (49.0)	185.7 (51.7)			N/A											

Abbreviations: BMI, body mass index; HbA1c, glycated hemoglobin; LD, loading dose; NLD, no loading dose.

### Risk of Bias and Certainty of Evidence

**Figure 2.**
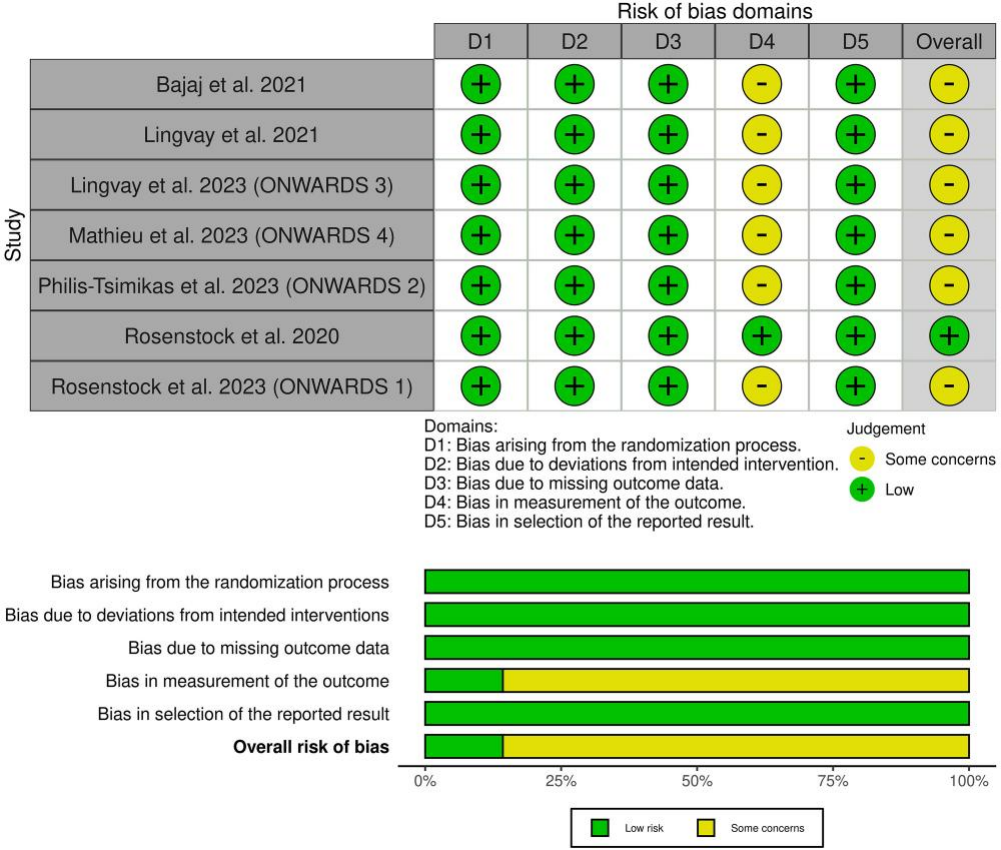
Quality assessment of risk of bias in the included trials. The upper panel presents a schematic representation of risks for specific types of biases of each of the studies in the review. The lower panel presents risks for the subtypes of biases of the combination of studies included in this review.

All of the included studies showed some concerns of detection bias due to the open-label design, except Rosenstock et al 2020, which was a double-blinded RCT [13] (Fig. 2, Supplementary Tables S2-S8) [21]. Certainty of evidence is demonstrated in a GRADE evidence profile (Table 3).

**Table 3.** GRADE evidence profile

Certainty assessment							Summary of findings				
Participants (studies) Follow-up	Risk of bias	Inconsistency	Indirectness	Imprecision	Publication bias	Overall certainty of evidence	Study event rates (%)		Relative effect (95% CI)	Anticipated absolute effects	
							With [Once-daily, long acting insulin]	With [Insulin Icodec]		Risk with [Once-daily, long acting insulin]	Risk difference with [Insulin Icodec]
HbA1C (%) change											
3182 (7 RCTs)	Not serious	Serious*^a^*	Not serious	Not serious	None	⨁⨁⨁◯ Moderate	1563	1619	—	The mean hbA1C (%) Change was −0.15	MD **0.15 lower** (0.24 lower to 0.06 lower)
Attainment of hemoglobin A1C target											
3182 (7 RCTs)	Not serious	Serious*^a^*	Not serious	Not serious	None	⨁⨁⨁◯ Moderate	685/1563 (43.8%)	833/1619 (51.5%)	RR 1.19 (1.04 to 1.37)	438 per 1000	**83 more per 1000** (from 18 more to 162 more)
Time with glucose in range (%)											
2594 (6 RCTs)	Not serious	Not serious	Not serious	Not serious	Strong association	⨁⨁⨁⨁ High	1269	1325	—	The mean time with Glucose in Range (%) was 4.06	MD **4.06 higher** (2.06 higher to 6.06 higher)
Body weight (kg) change											
3182 (7 RCTs)	Not serious	Serious*^a^*	Not serious	Not serious	Strong association	⨁⨁⨁⨁ High	1563	1619	—	The mean body Weight (kg) Change was 0.57	MD **0.57 higher** (0.45 higher to 0.7 higher)
Fasting plasma glucose (mg/dL) change											
3177 (7 RCTs)	Not serious	Not serious	Not serious	Very serious*^b^*	None	⨁⨁◯◯ Low	1560	1617	—	The mean fasting Plasma Glucose (mg/dL) Change was −1.10	MD **1.1 lower** (4.24 lower to 2.04 higher)
Safety outcomes—any adverse event											
3181 (7 RCTs)	Not serious	Not serious	Not serious	Not serious	None	⨁⨁⨁⨁ High	966/1563 (61.8%)	1057/1618 (65.3%)	RR 1.06 (1.01 to 1.12)	618 per 1000	**37 more per 1000** (from 6 more to 74 more)
Safety outcomes—any serious adverse event											
3181 (7 RCTs)	Not serious	Not serious	Not serious	Not serious	None	⨁⨁⨁⨁ High	133/1563 (8.5%)	128/1618 (7.9%)	RR 0.96 (0.76 to 1.20)	85 per 1000	**3 fewer per 1000** (from 20 fewer to 17 more)
Safety outcomes—any adverse event leading to withdrawal											
3181 (7 RCTs)	Not serious	Not serious	Not serious	Very serious*^b^*	None	⨁⨁◯◯ Low	16/1563 (1.0%)	26/1618 (1.6%)	RR 1.54 (0.84 to 2.82)	10 per 1000	**6 more per 1000** (from 2 fewer to 19 more)
Safety outcomes—all-cause mortality											
3181 (7 RCTs)	Not serious	Not serious	Not serious	Very serious*^b^*	None	⨁⨁◯◯ Low	8/1563 (0.5%)	11/1618 (0.7%)	RR 1.38 (0.56 to 3.41)	5 per 1000	**2 more per 1000** (from 2 fewer to 12 more)
Safety outcomes—injection-site reactions											
3181 (7 RCTs)	Not serious	Not serious	Not serious	Very serious*^b^*	None	⨁⨁◯◯ Low	33/1563 (2.1%)	45/1618 (2.8%)	RR 1.31 (0.85 to 2.02)	21 per 1000	**7 more per 1000** (from 3 fewer to 22 more)
Safety outcomes—hypersensitivity events											
3181 (7 RCTs)	Not serious	Not serious	Not serious	Very serious*^b^*	None	⨁⨁◯◯ Low	66/1563 (4.2%)	58/1618 (3.6%)	RR 0.86 (0.61 to 1.22)	42 per 1000	**6 fewer per 1000** (from 16 fewer to 9 more)

Abbreviations: CI, confidence interval; MD, mean difference; RCT, randomized controlled trial; RR, risk ratio.

^*a*^I^2^ > 50%.

^*b*^Wide confidence interval that does not exclude the risk of appreciable harm/benefit.

### Primary Outcome

**Figure 3.**
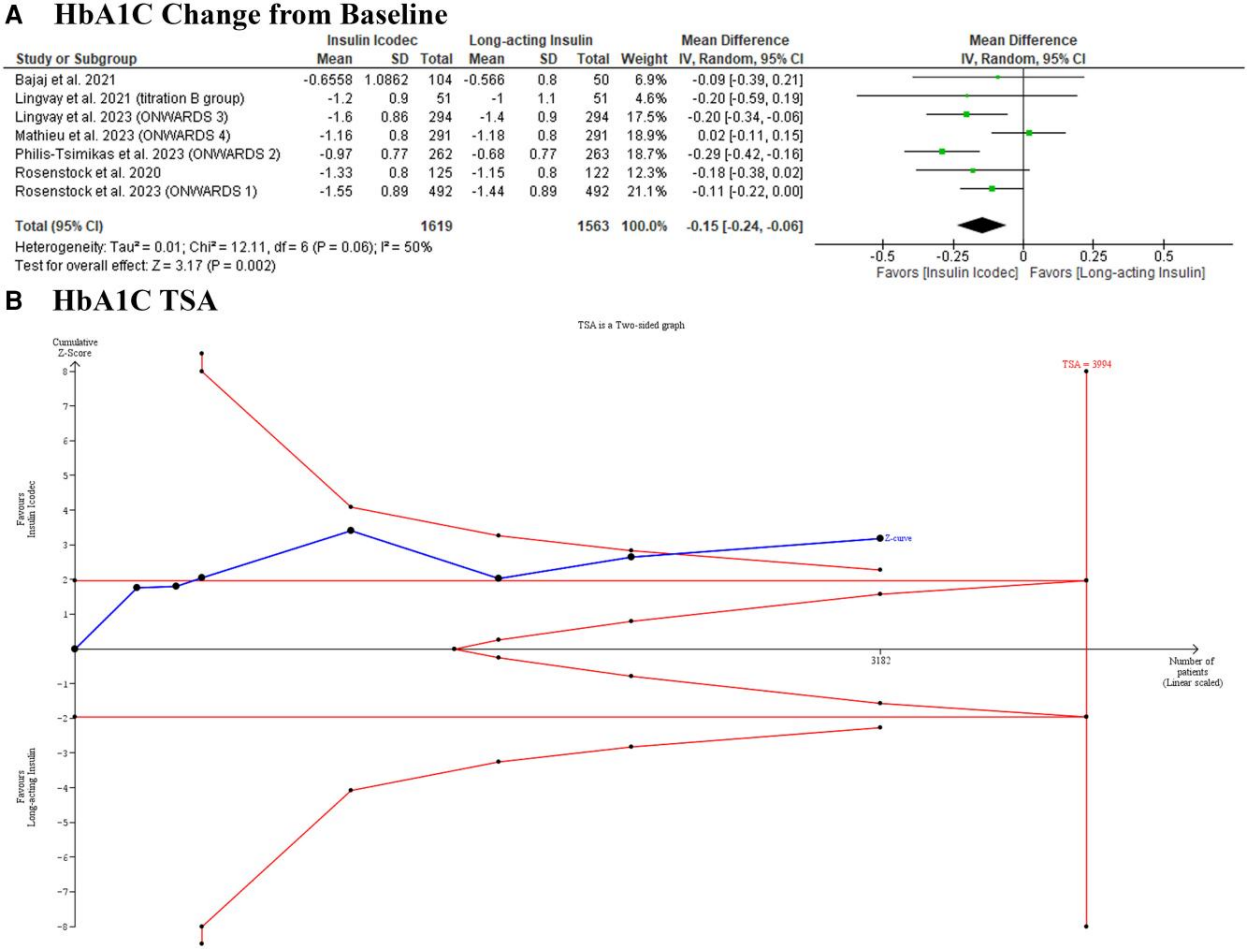
Forest plot and trial sequential analysis of the primary efficacy outcome (hemoglobin A1C change), (A) forest plot; (B) trial sequential analysis.

Abbreviations: CI, confidence interval; MD, mean difference.

Icodec was significantly associated with decreased HbA1C (MD: −0.15 with 95% CI [−0.24, −0.06], *P* = .002) (Fig. 3A). Pooled studies were heterogeneous (I^2^ = 50%, *P* = .06). Heterogeneity was resolved after excluding Mathieu et al 2023 (ONWARDS 4) and Philis-Tsimikas et al 2023 (ONWARDS 2) (I^2^ = 0%, *P* = .47), (I^2^ = 17%, *P* = .30), respectively (Supplementary Table S9) [21]. TSA showed that the available evidence surpassed the required information size and reached the trial sequential monitoring boundary, indicating robust conclusions (Fig. 3B).

The test for subgroup analysis was significant in loading dose (*P* = .02) (Supplementary Fig. S1) [21], with a greater reduction in HbA1C in patients who received a loading dose (MD: −0.28 with 95% CI [−0.41, −0.16], *P* < .00001), compared to those who did not receive a loading dose (MD: −0.10 with 95% CI [−0.19, −0.02], *P* = .02), and in the type of long-acting insulin in the control group subgroups (*P* = .007) (Supplementary Fig. S2) [21], insulin icodec more effective than insulin degludec (MD: −0.25 with 95% CI [−0.35, −0.15], *P* < .00001), compared to insulin glargine (MD: −0.08 with 95% CI [−0.15, −0.01], *P* = .04). However, it was not significant in the history of previous bolus insulin (*P* = .82) (Supplementary Fig. S3) [21] or trial phase subgroups (*P* = .87) (Supplementary Fig. S4) [21].

### Secondary Outcomes

#### Efficacy outcomes

**Figure 4.**
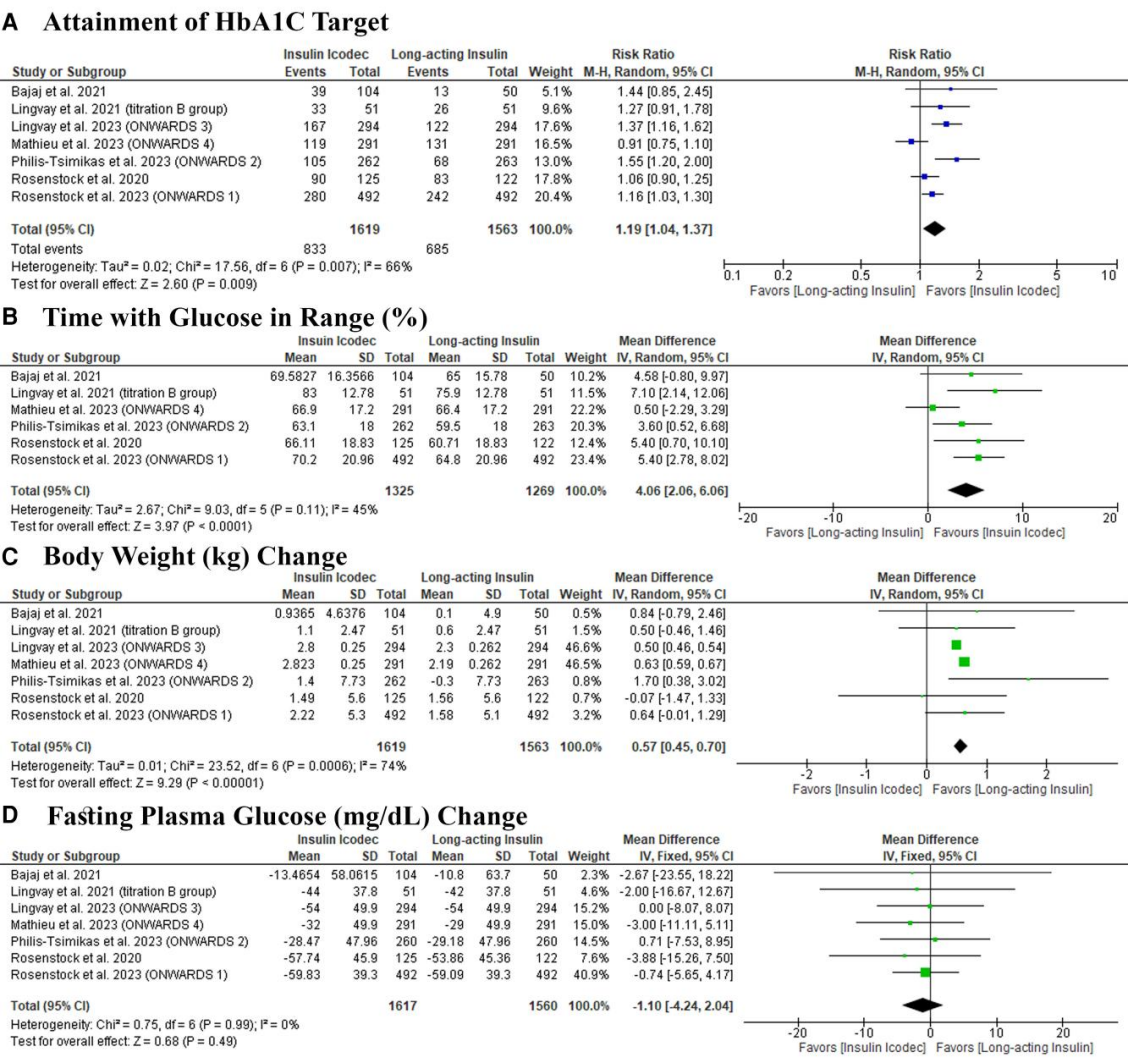
Forest plots of the secondary efficacy outcomes, (A) attainment of HbA1C target; (B) time with glucose in range (%); (C) body weight (kg) change; (D) fasting plasma glucose (mg\dl) change.

Abbreviations: CI, confidence interval; MD, mean difference; RR, risk ratio.

Icodec was significantly associated with an increased rate of HbA1C target attainment (RR: 1.19 with 95% CI [1.04, 1.37], *P* = .009) (Fig. 4A) and an increased percentage of TIR (MD: 4.06 with 95% CI [2.06, 6.06], *P* = .0001) (Fig. 4B). However, icodec was associated with increased body weight change (MD: 0.57 with 95% CI [0.45, 0.70], *P* = .00001) (Fig. 4C), with no difference between both groups regarding FPG change (MD: −1.10 with 95% CI [−4.24, 2.04], *P* = .49) (Fig. 4D). Pooled studies were homogenous in TIR (I^2^ = 45%, *P* = .11) and FPG change (I^2^ = 0%, *P* = .99). However, pooled studies were heterogeneous in HbA1C target attainment (I^2^ = 66%, *P* = .007) and body weight change (I^2^ = 74%, *P* = .0006). Regarding HbA1C target attainment, heterogeneity was best resolved after excluding Mathieu et al 2023 (ONWARDS 4) (I^2^ = 0%, *P* = .50). In body weight change heterogeneity was resolved after excluding Lingvay et al 2023 (ONWARDS 3) and Mathieu et al 2023 (ONWARDS 4), (I^2^ = 0%, *P* = .61) (I^2^ = 0%, *P* = .53), respectively (Supplementary Table S9) [21]. Further details on the actual change are outlined in Supplementary Table S10 [21].

TSA showed that the available evidence surpassed the required information size and reached the trial sequential monitoring boundary, indicating robust conclusions in HbA1C target attainment, TIR, and body weight change (Supplementary Figs. S5-S7) [21]. A test of subgroup analysis of all subgroups was insignificant, except for loading dose (*P* = .06) (Supplementary Fig. S8) [21], with a greater increase in HbA1C target attainment in patients who received a loading dose (MD: 1.97 with 95% CI [1.41, 2.77], *P* < .0001) compared to those who did not receive a loading dose (MD: 1.31 with 95% CI [1.00, 1.72], *P* = .05); type of long-acting insulin in the control group subgroups in HbA1C target attainment (*P* = .02) (Supplementary Fig. S9) [21], with insulin icodec more effective compared to insuin degludec (MD: 1.88 with 95% CI [1.47, 2.40], *P* < .00001) compared to insulin glargine (MD: 1.22 with 95% CI [0.93, 1.61], *P* = .15); and history of previous basal insulin in TIR (*P* = .05) (Supplementary Fig. S10) [21], which came in favor of patients with no history of previous basal insulin (MD: 5.70 with 95% CI [3.62, 7.78], *P* < .0001) compared to those with a history of previous basal insulin (MD: 2.41 with 95% CI [−0.04, 4.87], *P* = .05). The rest of the insignificant subgroup analysis can be found in Supplementary Figs. S11-S23 [21].

#### Safety outcomes

**Figure 5.**
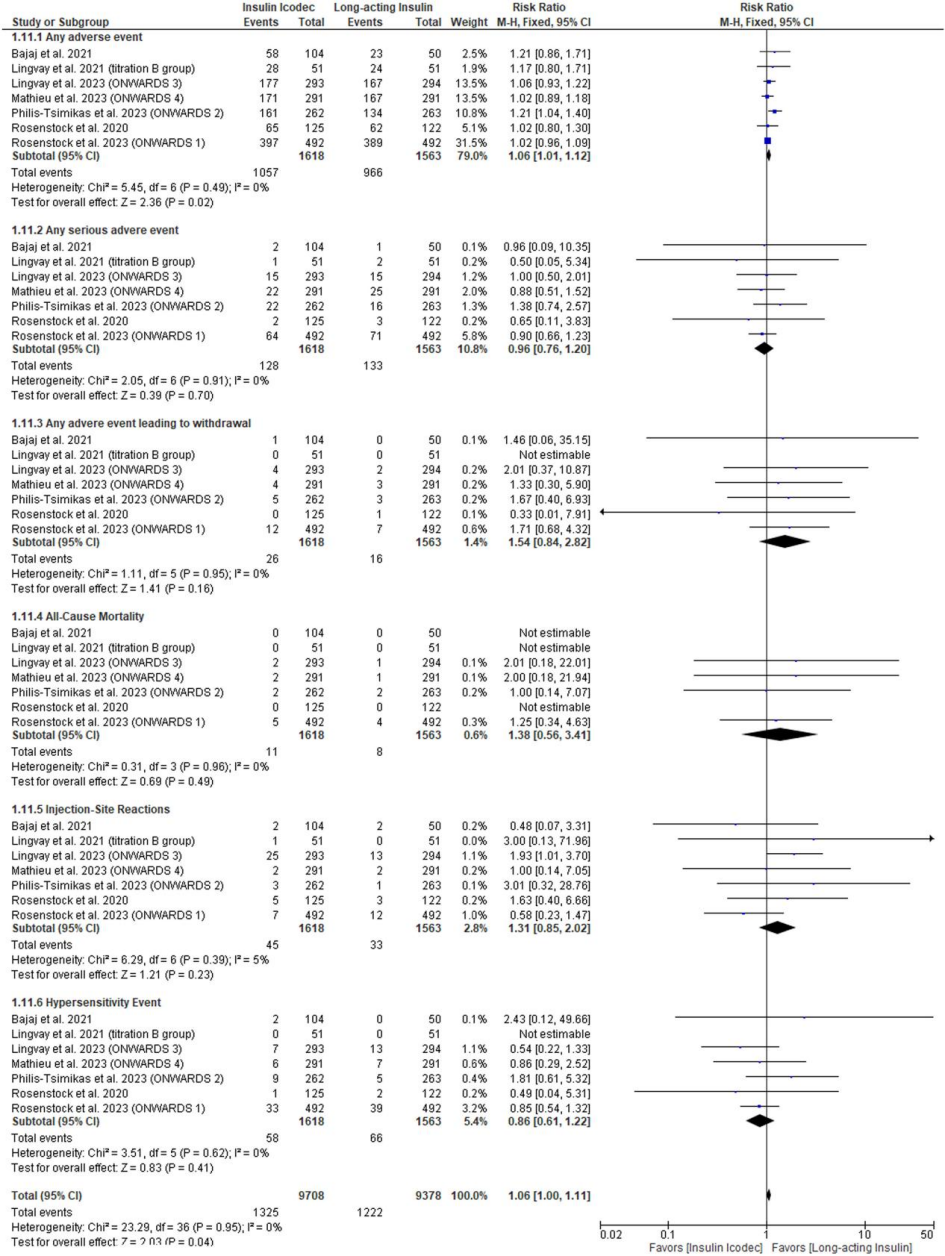
Forest plot of the safety outcomes.

Abbreviations: CI, confidence interval; RR, risk ratio.

Icodec was significantly associated with an increased rate of any adverse event incidence (RR: 1.06 with 95% CI [1.01, 1.12], *P* = .02). However, there was no difference between both groups regarding the incidence of any serious adverse event (RR: 0.96 with 95% CI [0.76, 1.20], *P* = .7), any adverse event leading to withdrawal (RR: 1.54 with 95% CI [0.84, 2.82], *P* = .16), all-cause mortality (RR: 1.38 with 95% CI [0.56, 3.41], *P* = .49), injection-site reactions (RR: 1.31 with 95% CI [0.58, 2.02], *P* = .23), or hypersensitivity events (RR: 0.86 with 95% CI [0.61, 1.22], *P* = .41) (Fig. 5). Pooled studies were homogenous across all safety outcomes (I^2^ = 0%).

Regarding hypoglycemic events, there was no difference between both groups in all grades of overall and hypoglycemic events, as shown in Supplementary Figs. S24 and S25 [21]. Pooled studies were homogeneous in severe hypoglycemia (level 3) (I^2^ = 0%, *P* = .49). However, pooled studies were heterogeneous in hypoglycemic alert (level 1) (I^2^ = 80%, *P* < .0001) and clinically significant hypoglycemia (level 2) (I^2^ = 61%, *P* = .02). Regarding hypoglycemic alert (level 1) heterogeneity was best resolved after excluding Mathieu et al 2023 (ONWARDS 4) (I^2^ = 0%, *P* = .66), and, in clinically significant hypoglycemia (level 2), heterogeneity was best resolved after omitting Philis-Tsimikas et al 2023 (ONWARDS 2) (I^2^ = 42%, *P* = .12) (Supplementary Table S9) [21].

## Discussion

In this systematic review and meta-analysis, insulin icodec significantly decreased HbA1C level, increased HbA1C target, and increased TIR percentage, with no difference in FPG changes compared to long-acting insulin. Additionally, insulin icodec, although significantly associated with body weight gain and an increase in any adverse event incidence, was not associated with an increased risk of other safety outcomes, including serious complications, complications prompting withdrawal, mortality due to any cause, injection-site events, and hypersensitivity reactions. Moreover, insulin icodec showed similar hypoglycemic events in terms of both incidence and severity compared to long-acting insulin groups. The reviewed body of evidence was prone to some concerns regarding potential detection bias resulting from the open-label design, except for 1 double-blinded RCT [13].

HbA1C is a product of a hemoglobin glycation reaction during which about 6% of the hemoglobin A interacts with blood glucose to form a glycoprotein with different fractions (of them, HbA1C is the most abundant). The latter has the same half-life as hematocytes (2 to 3 months); therefore, high levels of HbA1C are indicative of hyperglycemia during this period [28]. Reduction in the pathological HbA1C values reflects stable long-term hypoglycemic effects. We showed through the current meta-analysis that insulin icodec induced a significant decrease in HbA1C levels and an increase in HbA1C target attainment. This can be due to the pharmacokinetic and pharmacodynamic properties that insulin icodec exhibits, including strong albumin binding potency and reduced insulin receptor affinity, resulting in slow insulin receptor-mediated clearance and inactive albumin-bound aggregates of insulin, which ensures slow but continuous metabolic action, manifesting as consistent glucose-lowering effects achieved throughout the week at clinically relevant doses [9, 29].

Moreover, since previous experiences with once-weekly glucagon-like peptide-1 receptor agonist dosing have demonstrated a considerable reduction in the risk of nonadherence when compared to once-daily dosing [30], the same advantage is likely to be seen with once-weekly insulin as it did not increase the risk of developing hypoglycemia; however, it was also associated with increased body weight, which may influence the patient's adherence. Thus, insulin icodec can enhance treatment adherence, leading to more optimal long-term control of blood glucose levels. Notably, treatment satisfaction in T2D patients was shown to be better improved with switching to insulin icodec than to insulin degludec [31].

Another possible reason for the glycemic control with insulin icodec is the pharmacological stability offered by the once-weekly administration mode, which can narrow or even avoid day-to-day variation in glucose-lowering effects observed with long-acting insulin such as glargine [32]. Day-to-day variation assessed by continuous glucose monitoring was found to be negatively impacted by the durability of basal insulin; thus, lower variations were seen in insulin with more prolonged absorption time [33]. Hence, patients with large interday glycemic variability are likely at higher risk of nonimprovement in HbA1C levels and vice versa [34]. Knowing that insulin icodec has high durability and retention in blood, this property may contribute to the observed ameliorations in pathological glycated hemoglobin parameters. Moreover, increasing the duration of action of insulin analogs can reduce the intra-individual variability in glucose-lowering effects [35], which may also participate in better quantitative and qualitative corrections of HbA1C.

Similarly, icodec had positive effects on the other glycemic control metric (TIR). The increase in TIR percentage reflects the potential of a once-weekly insulin regimen in decreasing blood glucose to approach the desired target range. The International Consensus recommends a mean TIR of >70% and more clinical benefit with each 5% increase in TIR [36]. Icodec achieved a 4.06% increase in TIR compared with once-daily insulin, which supports its superior benefit in reaching clinically significant glycemia changes. However, there was no superiority between the 2 regimens in modifying FPG, perhaps because, at fasting, both achieve similar inhibition of gluconeogenesis and insulin-dependent glucometabolic pathways.

In contrast, patients treated with insulin icodec developed higher body weight gain compared to controls. Insulin icodec has an affinity to albumin that is 9.5-fold greater than that of long-acting insulin; therefore, it leads to considerably slower and longer activation of the insulin receptors [9]. From this, we hypothesize that insulin icodec therapy may trigger a sort of “basal” lipogenesis, adipogenesis, and glycogenesis after reaching the steady state. Especially, insulin icodec exerts continuous stimulation of insulin-dependent uptake of glucose [9], which is known to enhance lipogenesis in hepatocytes and adipocytes via the upregulation of several lipogenic pathways (ie, SREBP-1 and PPARγ-associated pathways) [37]. Therefore, continuous insulin-dependent glucose uptake provided by insulin icodec may lead to continuous activation of cellular lipogenic and, therefore, anabolic pathways. At the same time, the maintained effects of insulin icodec may ensure a sustainable insulin-mediated suppression of lipolysis, which is a major effect of insulin on fat metabolism [38], most likely favoring a cellular shift toward the anabolic state, eventually resulting in weight gain.

In the meta-analysis of safety outcomes, despite the higher risk for any adverse event observed with insulin icodec, the rates of serious adverse events and hypoglycemia episodes were comparable with those of control groups, indicating overall a noninferior safety profile. Generally, the risk of hypoglycemia is related to prandial and premixed bolus insulin therapy rather than basal insulin [39, 40]. Since insulin icodec has slow and progressive action on the insulin receptors, it has a low potential to increase the likelihood of substantial acute or delayed glucose movement toward cells (about the administration timing) with concomitant inhibition of glucagon and catecholamines, which are the main required conditions for insulin therapy associated hypoglycemia at a normal physical state. Thereby, mechanistically speaking, the probability that at standard dosing insulin icodec causes excessive acute glucogenic effects leading to hypoglycemia events compared to once-daily insulin seems to be low.

### Strengths and Limitations

This is the first comprehensive systematic review and meta-analysis that included phase III trials comparing the efficacy and safety of insulin icodec and long-acting insulin in T2D patients. After searching several databases, we included 7 peer-reviewed RCTs published in international journals reporting data of 3183 T2 D patients. We used TSA to evaluate the validity of the pooled data and determine if the sample size of the current meta-analysis was sufficient to draw firm conclusions [26]. We also used subgroup analysis to resolve the heterogeneity of pooled data and estimate the outcomes in different groups. Additionally, the current study is the first to apply the GRADE framework for evaluating the quality of evidence regarding the efficacy and safety of insulin icodec in comparison to long-acting insulin therapy in T2D.

However, our results were challenged by important limitations. First, we have tried to investigate all outcomes in each available subgroup; the available RCTs excluded many interesting subgroups, such as patients with chronic renal failure or liver failure. Second, Lingvay et al evaluated 3 alternative insulin icodec titration algorithms based on weekly insulin dosage adjustment and prebreakfast self-monitoring of blood glucose [12]. Nevertheless, we included only titration B group in our analysis because it is the same titration regimen used in the control glargine group (28 unit/week adjustment with a prebreakfast glycemia target of 80-130 mg/dL) as recommended by the American Diabetes Association [41]. Third, 6 RCTs [12, 14-18] were underpowered by the open-label design, which was likely chosen due to safety precautions as stated by Mathieu et al [15].

Another drawback in our study is the inclusion of studies with varying follow-up durations, which complicates direct comparisons of the change in HbA1c However, despite this inherent heterogeneity, a noteworthy observation emerged: despite diverse follow-up periods ranging from 5 weeks to 31 weeks, the outcomes consistently demonstrated comparable results. This intriguing finding suggests a certain level of robustness and uniformity in the intervention's effects across these differing timeframes. This consistency lends support to the reliability and potential generalizability of our findings, suggesting that the intervention's impact remains relatively stable irrespective of the duration of follow-up periods.

Finally, in 1 study [13], flash glucose monitoring was used instead of continuous glucose monitoring to monitor TIR, which may provide less accurate measurements. Nonetheless, all the remaining studies reported using double-blinded continuous glucose monitoring.

### Implications for Clinical Practice

T2D patients who are candidates for basal insulin are those whose glycemic control targets cannot be achieved with oral hypoglycemic drugs, and, therefore, they require the introduction of insulin therapy either as an adjuvant or switching treatment strategy [42]. There are several challenges of insulin therapy in this category of patients; among them are frequently reported managing time concerns by both patients and physicians, lack of confidence regarding self-managing abilities, perception of insulin regimen as complex therapy, and needle fear and/or anxiety [42, 43]. These barriers can lead to significant delays and/or interruptions in insulin administration, exposing patients to higher risks of unbalanced diabetes complications [42].

The simplicity and low injection frequency of an insulin icodec-based regimen may reduce the main patient-related concerns regarding injection timing, treatment complexity, the necessity of high vigilance/understanding of treatment protocol, misuse, needle phobia, and the experience of being daily self-injected. This regimen can also facilitate patients’ education, monitoring, and treatment adjustment by physicians, enhancing their likelihood to initiate/intensify insulin therapy when needed, ultimately decreasing the phenomenon of clinical inertia. The latter, defined as failure to start or intensity insulin therapy when indicated, seems to be more common among less experienced physicians [43]. This makes the more easy-to-establish hebdomadal insulin icodec regimen an interesting strategy to antagonize the hesitancy of prescribing insulin in the context of T2D. Additionally, patients with T2D are mostly comorbid, receiving different chronic treatments. Consequently, once-weekly insulin icodec can lower the polypharmacy burden among these patients, which would at the same time favor their acceptance of insulin therapy.

On the other hand, patients treated with insulin icodec are more susceptible to gaining weight, probably by hyperstimulated lipogenesis/adipogenesis, which would limit the benefit of this therapeutic option. Such adverse effect is concerning and should be further evaluated before any clinical use of insulin. Thus, since the majority of T2D patients suffer from overweight or obesity, even a modest increase in their body fat can be deleterious and can increase cardiovascular risks. Therefore, patients with severely high body mass index may not be the best candidates for insulin icodec. Nonetheless, less obese patients can be educated on a possible moderate weight gain and, as a result, can have a close monitoring of their weight. This risk can be an occasion to remind diabetic patients of the benefits of weight loss in lowering the incidence of cardiovascular events. As metformin is known for its weight loss-promoting action [44], its addition to the insulin icodec regimen may antagonize the latter's adipogenic potentials.

### Implications for Future Research

Despite the validity of the current evidence, several issues remain to be investigated. First, insulin icodec is yet to be investigated in type I diabetes. Second, the safety of insulin icodec across the different degrees of liver and renal failure is still to be reported with an ongoing trial in patients with hepatic dysfunction [45]. Third, the hypoglycemic effect of insulin icodec during exercise or under the effect of other clinical conditions is still unknown, warranting real-world setting studies [46]. Finally, despite not being significantly associated with hypoglycemia, managing any hypoglycemic cases by dose adjustment would wait for a week, and the patient may need to increase carbohydrate intake to compensate [46]. This concern should be further investigated.

## Conclusion

Once-weekly insulin icodec is a potential game-changer in T2D management, and it can be used instead of once-daily long-acting regimens without compromising the overall efficacy and safety profile of basal insulin therapy in T2D patients. This could enhance treatment acceptance, satisfaction, and adherence by patients while facilitating their management by physicians. However, when applying this regimen, strict monitoring and anticipated precautions should be retained, notably regarding the risk of weight gain, hypoglycemia, and the other adverse events of insulin therapy.

## Data Availability

Some or all datasets generated during and/or analyzed during the current study are not publicly available but are available from the corresponding author on reasonable request.
